# Adolescent with Rhabdomyolysis due to Undiagnosed Hypothyroidism

**DOI:** 10.1155/2011/670673

**Published:** 2011-10-11

**Authors:** Raquel Farias Moeller, Nassim Zecavati, Rosa Sherafat-Kazemzadeh, Shoshana Aleinikoff, Wolfgang Rennert

**Affiliations:** ^1^Department of Pediatrics, Georgetown University Hospital Medical Center, Washington, DC, USA; ^2^Division of Neurology, Georgetown University Hospital Medical Center, Washington, DC, USA; ^3^Division of Endocrinology and Metabolism, Georgetown University Hospital Medical Center, Washington, DC, USA

## Abstract

Exercise-induced rhabdomyolysis has been described in military recruits, trained athletes and daily runners. Statin use, quail ingestion, infection by Epstein-Barr virus (EBV), and hypothyroidism, though rare, are risk factors for the development of rhabdomyolysis. We describe the case of a 15-year-old female who presented with myalgias, weakness, and pigmenturia following marching band practice. Laboratory tests confirmed an elevated creatine kinase (CK) level as well as a profound hypothyroid state. Muscle biopsy revealed severe muscle necrosis and myositis. Treatment with levothyroxine resulted in obtaining an euthyroid state and regain of muscle strength as well as decrease in CK levels. Although rare, hypothyroidism should be considered as a potential cause of rhabdomyolysis in pediatric patients undergoing a myopathy workup.

## 1. Introduction

Rhabdomyolysis is a condition characterized by leakage of muscle-cell content into the circulation [1]. Symptoms include myalgias, edema, weakness, and pigmenturia caused by myoglobinuria. Diagnosis of rhabdomyolysis is based on elevated serum CK levels of more than 1000 U/L [2]. Trauma, exertion, muscle hypoxia, genetic defects, infections, body-temperature changes, metabolic and electrolyte disorders, drugs, and toxins are causes of rhabdomyolysis [1]. Hypothyroid patients may present with myopathy and mild elevation of CK levels; however, overt rhabdomyolysis is extremely rare, and few cases have been described [3–10].

We report the case of an adolescent who presented with weakness, myalgias, and pigmenturia after attending marching band practice and was later found to be profoundly hypothyroid. A thorough literature review was done, and only eight cases of hypothyroid-induced rhabdomyolysis have been reported.

## 2. Case

A 15-year-old Caucasian female was admitted to the general pediatric service at our institution with a three-week history of progressive myalgias and profound proximal muscle weakness of the bilateral lower extremities. She noted dark urine two weeks prior to presentation and reported weight gain, fatigue, and leg swelling of six months' duration. She denied taking any medications. Her family history was remarkable for adult onset hypothyroidism in her mother. 

On physical examination, the patient weighed 100 kg (+2.41 SD) and was 170 cm in height (+1.24 SD) with stable vital signs. She had bilateral, knee-level, nonpitting edema without periorbital edema or goiter. Her neurological examination revealed intact cranial nerves, bilateral symmetric proximal muscle weakness (3/5) in the quadriceps, hamstrings, hip flexors, and extensors, and intact sensation to light touch, temperature, and proprioception. Deep tendon reflexes were absent in all four extremities. Both thighs were tender to palpation. She had Tanner 4 breasts and pubic hair development. The remainder of her physical and neurological examination was within normal limits. 

Laboratory results included normal hemoglobin, electrolytes, and renal function. Her creatine kinase (CK) was elevated at 34724 IU/L (26–140) and was accompanied by elevation of transaminases and myoglobinuria. Thyroid studies revealed the following: thyroid stimulating hormone (TSH) 77.2 mIU/mL (0.35–5.5), free thyroxine (fT4) 0.17 ng/dL (0.58–1.64), and anti-thyroid peroxidase (anti-TPO) antibody 162 IU/mL (<35) (Table 1). Electromyography/nerve conduction Studies showed short duration, low amplitude motor units with an early recruitment pattern, 2+ fibrillations, and 2+ positive sharp waves in all muscle samples, consistent with a myopathic process. The patient was diagnosed with rhabdomyolysis in the setting of severe hypothyroidism unmasked by moderate exertion. She was treated with aggressive intravenous fluid replacement (4 liters/day of 0.45% saline) and strict bed rest. Levothyroxine replacement of 75 mcg/day was started on day two of hospitalization, and on day seven, the dose was increased to 100 mcg/day.

**Figure 1:**
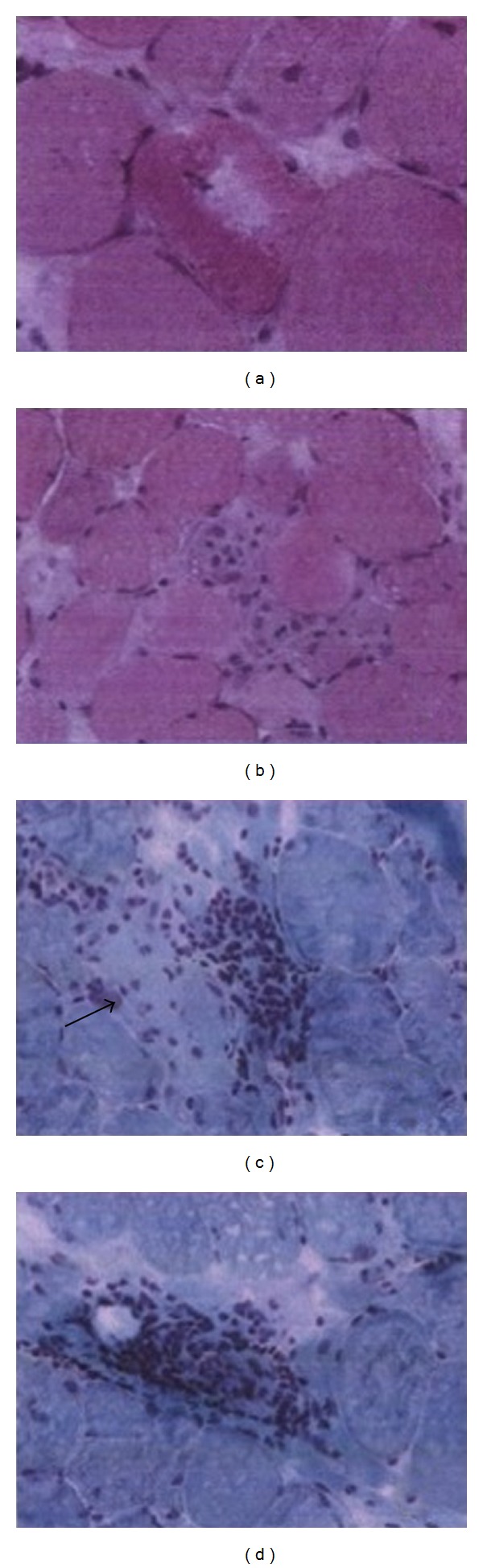
Muscle biopsy: (a) vacuolated fibers, (b) necrotic fibers, (c and d) endomysial and perivascular inflammation as well as necrotic fibers (black arrow).

Despite rapid clinical improvement, our patient continued to have elevated CK values (Table 1) warranting further investigation for other etiologies of rhabdomyolysis, including testing for infection with Epstein-Barr virus (EBV) and cytomegalovirus (CMV), both of which were negative. She did not report using (HMG)-CoA reductase inhibitors, which have been associated with rhabdomyolysis [9]. On the second week of hospitalization, a muscle biopsy was performed to rule out metabolic etiologies predisposing to rhabdomyolysis. The biopsy revealed severe myonecrosis and inflammation (Figure 1). It ruled out congenital metabolic myopathies such as myophosphorylase deficiency (McArdle's disease), carnitine deficiency syndromes, or defective beta-oxidation enzymes. The slow decline in CK values as well as persistent weakness was consistent with severe muscle injury facilitated by profound hypothyroidism and moderate exercise (Figure 1). 

Our patient continued to improve clinically, her CK levels decreased to 22737 IU/L (Table 1), and on day fourteen, she was discharged on levothyroxine at a daily dose of 150 mcg. At her follow-up visit two weeks after discharge, the patient remained clinically well with improved fatigue. She experienced a 4.8 kg weight loss but retained mild weakness in her hip flexors and extensors (4/5 strength). Approximately two months after presentation, her thyroid function tests had normalized and her CK level had declined to <6,000 IU/L (Table 1).

## 3. Discussion

We describe a case of overt rhabdomyolysis with severe muscle necrosis due to undiagnosed hypothyroidism, following moderate physical exertion. Myopathy is a common presentation of hypothyroidism and may be accompanied by asymptomatic or mild to moderate CK elevation, usually less than ten times the upper limit of normal [6]. To our knowledge, there have been eight case reports of hypothyroid-induced rhabdomyolysis in the English literature [3–10]; three have been reported in children or young adults [5, 7, 10]. Although the CK level does not correlate with the severity of the myopathic process [8], the CK elevation in our patient was profound. Literature review indicates that this patient's CK value is the highest documented value in a patient with hypothyroid-induced rhabdomyolysis [3–10].

Rhabdomyolysis is characterized by the leakage of muscle-cell contents, including electrolytes, myoglobin, and other sarcoplasmic proteins (e.g., creatine kinase, aldolase, lactate dehydrogenase, alanine aminotransferase, and aspartate aminotransferase) into the circulation [1]. Common, but not universal, presenting symptoms include myalgias, muscle tenderness, edema, and weakness [2] as well as dark, “cola-colored” urine, all of which were present in our patient. Acute renal injury associated with myoglobinuria, reported in 13 to 50% of cases, is the most serious complication of rhabdomyolysis [1]. Early, aggressive hydration is warranted to prevent acute tubular necrosis and maintain good urine output. With aggressive hydration, our patient maintained adequate renal function despite profound muscle necrosis and myoglobinuria. Although renal failure is not uncommon in rhabdomyolysis, the outcome is usually favorable [7].

The exact pathogenesis of hypothyroid-induced rhabdomyolysis is not completely elucidated [4, 6, 8]. It has been proposed that thyroxine deficiency leads to the following changes: muscle fibers' switch from fast twitching type II to slow twitching type I fibers, there is deposition of glycosaminoglycans, poor contractility of actin-myosin units, low myosin ATPase activity, and low ATP turnover in skeletal muscle [5], as well as hypoperfusion of muscular vessels and muscle tissue hypoxia with low muscle energy stores [8].

Rhabdomyolysis is commonly seen after vigorous exercise, and patients with hypothyroidism may be particularly vulnerable to this disease process. We therefore propose a high level of suspicion for the diagnostic evaluation of myopathy in a hypothyroid patient with nonspecific complaints of fatigue, myalgias, or generalized weakness. CK levels mostly correlate with the extent of muscle injury rather than the severity of hypothyroidism. Treatment with levothyroxine and aggressive rehydration should be promptly instituted. In summary, rhabdomyolysis should be entertained as a possible diagnosis in the hypothyroid patient who develops myalgias and weakness even after mild exertion.

## Figures and Tables

**Table 1:** Patient's laboratory values.

Variables	Day 1	Day 2	Day 3	Day 4	Day 10	Day 14	Day 40	Day 70
CK (26–140 IU/L)	34724	23731	26188	29060	25844	22737	9212	5545
LDH (91–180 IU/L)	1150	764						
TSH (0.35–5.500 mIU/mL)	77.2			60	65		10.9	2.38
FT4 (0.58–1.64 ng/dL)	0.17			0.31	0.63		1.18	1.09
T4 (6.09–12.23 mcg/dL)	2						8.2	8.2
Urine myoglobin (<0.025 mcg/mL)	137			40	31			
Serum myoglobin (<30 mcg/mL)	4780							
Aldolase (3.4–8.6 U/L)	347	291						
AST (10–31 IU/L)	931	733	571	667	547	565	416	89
ALT (7–35 IU/L)	820	699	602	588	514	495	510	109
BUN (6–19 mg/dL)	7	6	4	3	10	11	8	7
Creatinine (0.5–1.04 mg/dL)	0.4	0.3	0.4	0.4	0.4	0.4	0.4	0.5
Anti-TPO antibody (<35 IU/mL)	162							

CK: creatine kinase, TSH: thyroid-stimulating hormone, T4: thyroxine, FT4: free thyroxine, Anti-TPO: anti-thyroid peroxidase, LDH: lactic dehydrogenase, AST: aspartate aminotransferase, ALT: alanine aminotransferase, BUN: blood urea nitrogen.
